# Proteins function for anti-senescence and monoclonal proliferation of ATL cells

**DOI:** 10.1186/1742-4690-8-S1-A127

**Published:** 2011-06-06

**Authors:** Ratiorn Pornkuna, Shigeki Takemoto, Koji Uzawa, Kazuki Morita, Fumio Kawano

**Affiliations:** 1 Institute for Clinical Research, National Hospital Organization Kumamoto Medical Center, Kumamoto-City, Kumamoto, 860-0008, Japan; 2 Department of Hematology, National Hospital Organization Kumamoto Medical Center, Kumamoto-City, Kumamoto, 860-0008, Japan; 3 Research Laboratories, KYOWA MEDEX CO., LTD., Shizuoka, Japan

## Background

ATL is a highly aggressive leukemia/lymphoma which was first proposed as a new disease entity in 1977. The long clinical latency and low incidence of ATL indicate that some genetic changes are involved in malignant transformation and monoclonal expansion of HTLV-1-infected cells. Monoclonal proliferation of HTLV-1-infected cells is observed in a part of the virus carriers, who are considered to be the high risk group for development of ATL [1].

We reviewed the process of senescence and how proteins allow the monoclonal proliferation of ATL cells.

## Results

Constitutive activation of STAT as well as functional impairment and stabilization of p53 protein found in the PBMCs of ATL patients are supposed to be one base for ATL development [2,3]. In addition to deletion and/or methylation of the p16INK4A gene, they suggest the inhibition of senescence in ATL cells.

Furthermore, the soluble form of CD30 (sCD30) is elevated in serum of ATL patients and correlates with the aggressiveness of ATL [4] (Our observation) and it is useful marker which indicates the intervention of initiation therapy (Our observation) suggesting that sCD30 allows the proliferation and survival of ATL cells.

## Conclusions

Not only the inhibition of senescence by impaired function or constitutive activation of proteins in ATL cells, but also immune regulation by soluble proteins in serum of HTLV-1-infected patients must be required for the progression of ATL.

## References

[B1] Takemoto S Matsuoka M Yamaguchi K Takatsuki K A novel diagnostic method of adult T cell leukemia: Monoclonal integration of human T cell lymphotropic virus type I provirus DNA detected by inverse polymerase chain reaction Blood 1994 84 3080 5 7949180

[B2] Takemoto S Mulloy JC Cereseto A Migone TS Patel BKR Matsuoka M Yamaguchi K Takatsuki K Kamihira S White JD Leonard WJ Waldmann T Franchini G Proliferation of adult T cell leukemia/lymphoma cells is associated with the constitutive activation of JAK/STAT proteins Proc Natl Acad Sci USA 1997 94 13897 902 10.1073/pnas.94.25.13897 9391124 PMC28404

[B3] Takemoto S Trovato R Cereseto A Nicot C Kislyakova T Casareto L Waldmann T Torelli G Franchini G p53 stabilization and functional impairment in the absence of genetic mutation or the alteration of the p14ARF MDM2 loop in ex vivo and cultured adult T cell leukemia/lymphoma cells Blood 2000 95 3939 44 10845931

[B4] Nishioka C Takemoto S Kataoka S Yamanaka S Moriki T Shoda M Watanabe T Taguchi H The serum level of soluble CD30 correlates with the aggressiveness of adult T cell leukemia/lymphoma Cancer Sci 2005 96 810 815 10.1111/j.1349-7006.2005.00106.x 16271075 PMC11158340

